# Microalgae-Derived Vesicles: Natural Nanocarriers of Exogenous and Endogenous Proteins

**DOI:** 10.3390/plants14152354

**Published:** 2025-07-31

**Authors:** Luiza Garaeva, Eugene Tolstyko, Elena Putevich, Yury Kil, Anastasiia Spitsyna, Svetlana Emelianova, Anastasia Solianik, Eugeny Yastremsky, Yuri Garmay, Elena Komarova, Elena Varfolomeeva, Anton Ershov, Irina Sizova, Evgeny Pichkur, Ilya A. Vinnikov, Varvara Kvanchiani, Alina Kilasoniya Marfina, Andrey L. Konevega, Tatiana Shtam

**Affiliations:** 1 Petersburg Nuclear Physics Institute Named by B.P. Konstantinov of National Research Centre “Kurchatov Institute”, Orlova roscha 1, Gatchina 188300, Russia; garaeva_laa@pnpi.nrcki.ru (L.G.); tolstyko_ea@pnpi.nrcki.ru (E.T.); lena.put23@gmail.com (E.P.); kil_yv@pnpi.nrcki.ru (Y.K.); spitsyna_as@pnpi.nrcki.ru (A.S.); emelyanova_ss@pnpi.nrcki.ru (S.E.); solianik_am@pnpi.nrcki.ru (A.S.); yuri.from.spb@gmail.com (Y.G.); varfolomeeva_ey@pnpi.nrcki.ru (E.V.); ershov_ao@pnpi.nrcki.ru (A.E.); sizova_ia@pnpi.nrcki.ru (I.S.); pichkur_eb@nrcki.ru (E.P.); kvanchiani_vv@pnpi.nrcki.ru (V.K.); konevega_al@pnpi.nrcki.ru (A.L.K.); 2 National Research Center “Kurchatov Institute”, Akademika Kurchatova pl. 1, Moscow 123182, Russia; e.yastremsky@gmail.com; 3 Laboratory of Cell Protection Mechanisms, Institute of Cytology of Russian Academy of Sciences, Tikhoretsky Ave. 4, St. Petersburg 194064, Russia; elpouta@yahoo.com; 4 Laboratory of Molecular Neurobiology, School of Life Sciences and Biotechnology, Shanghai Jiao Tong University, Shanghai 200240, China; i.vinnikov@sjtu.edu.cn; 5 Faculty of Pharmacy and Nutrition, UCAM, Universidad Católica de Murcia, 30107 Murcia, Spain; 6 Basic Department “Molecular and Structural Biology”, Peter the Great St. Petersburg Polytechnic University, Politehnicheskaya 29, St. Petersburg 195251, Russia

**Keywords:** microalgae, plant-derived vesicles, exosomes, drug delivery systems, cryo-EM, proteomics, *Chlamydomonas reinhardtii*, *Parachlorella kessleri*

## Abstract

Extracellular vesicles (EVs), nanoscale membrane-enclosed particles, are natural carriers of proteins and nucleic acids. Microalgae are widely used as a source of bioactive substances in the food and cosmetic industries and definitely have a potential to be used as the producers of EVs for biomedical applications. In this study, the extracellular vesicles isolated from the culture medium of two unicellular microalgae, *Chlamydomonas reinhardtii* (Chlamy-EVs) and *Parachlorella kessleri* (Chlore-EVs), were characterized by atomic force microscopy (AFM), cryo-electronic microscopy (cryo-EM), and nanoparticle tracking analysis (NTA). The biocompatibility with human cells in vitro (HEK-293T, DF-2 and A172) and biodistribution in mouse organs and tissues in vivo were tested for both microalgal EVs. An exogenous therapeutic protein, human heat shock protein 70 (HSP70), was successfully loaded to Chlamy- and Chlore-EVs, and its efficient delivery to human glioma and colon carcinoma cell lines has been confirmed. Additionally, in order to search for potential therapeutic biomolecules within the EVs, their proteomes have been characterized. A total of 105 proteins were identified for Chlamy-EVs and 33 for Chlore-EVs. The presence of superoxide dismutase and catalase in the Chlamy-EV constituents allows for considering them as antioxidant agents. The effective delivery of exogenous cargo to human cells and the possibility of the particle yield optimization by varying the microalgae growth conditions make them favorable producers of EVs for biotechnology and biomedical application.

## 1. Introduction

The existence of multivesicular bodies (MVBs) in plant cells was first mentioned in two independent papers in 1967 demonstrating that fusion of MVBs with the plasma membrane might result in the release of small vesicles into the extracellular space in fungi and higher plants [1,2]. The MVB-mediated secretion pathway was later confirmed in studies on barley leaves affected by powdery mildew fungus [3]. Over the past decade, the annual number of studies devoted to the biogenesis and functionality of plant extracellular vesicles (PEVs) has been growing. Of particular interest is the prospect of using PEVs in biomedicine, namely as natural carriers of endogenous and exogenous biomolecules to human cells. Thus, plant vesicles possess anti-inflammatory [4,5,6], regenerative [7,8,9], and antioxidant [7,10,11] properties. The successful delivery of exogenous therapeutic biomolecules with the plant-derived extracellular vesicles has also been widely discussed in the literature [6,12,13,14,15,16,17]. For example, delivery of siRNA or the antitumor drug doxorubicin by ginger vesicles has been demonstrated [12,18]. In addition, delivery of various therapeutic agents, including the therapeutic protein HSP70, by grapefruit vesicles or nanovectors has been shown in a number of studies [19,20]. Therapy of malignant neoplasms with an immunostimulatory strategy based on heat shock proteins, including HSP70, is a promising area of research [21]. A number of studies indicate the potential of using HSP70-based vaccines [21,22]. A significant increase in the efficiency of accumulation of functionally active HSP70 in tumor cells when delivered as part of grapefruit extracellular vesicles has been demonstrated in vitro and in vivo [16,20]. Moreover, the efficiency of therapy with HSP70-loaded grapefruit extracellular vesicles has been demonstrated in an animal model of colon cancer [20].

The choice of vesicle source is one of the key issues when using PEVs for biomedical applications. Algae are considered one of the promising producers of extracellular vesicles. Interestingly, the existence of exosome-like particles in algal cells was first mentioned in 1963, much earlier than for currently well-studied exosomes of animal origin, and somewhat earlier than for higher plants and fungi [23]. Recently, extracts of various algae have been considered for biomedical and cosmetic applications [24,25,26]. For instance, in vitro experiments performed by Gunathilaka et al. demonstrated a promising antidiabetic potential for extracts of the red algae *Gracilaria edulis* [25]. In another study, extracts of the unicellular diatom *Phaeodactylum tricornutum* reduced the oxidative damage of proteins caused by irradiation, indicating their possible use to protect epidermal cells from ultraviolet radiation [27]. The extracts of Korean brown algae *Codium fragile* or *Sargassum fusiforme* [26] and *Ecklonia cava* [28] contributed to the reduction in melanin synthesis, suggesting that they can be used as cosmetic, bleaching agents as well as for hyperpigmentation control. Extracts from the unicellular alga *Chlorella* demonstrated antioxidant and anti-inflammatory activities, while also containing phytochemical compounds and minerals, such as Cu, Se, Zn, Fe [29]. Potentially strong anti-inflammatory properties have also been revealed in the green alga *Chromochloris zofingiensis* [30].

Since a number of algae extracts have shown therapeutic potential, it is assumed that the vesicles produced by algae can carry biomolecules that retain similar therapeutic properties, although a practical testing of this assumption has been reflected in a few studies. Thus, extracellular vesicles obtained from the Korean algae *Codium fragile* and *Sargassum fusiforme* contributed to a decrease in melanin synthesis with the same efficiency as extracts [17]. In another work, the authors suggested that seaweed vesicles, as well as extracts, may contain miRNAs and antioxidants that can inhibit the proliferation of cancer cells [24].

In biotechnology and biomedicine applications, the unicellular algae could become one of the most promising producers of vesicles, since they can be grown in bioreactors. By varying their growth conditions, the yield and composition of the produced particles can be improved. Thus, the physical characteristics and yield efficiency of PEVs derived from 18 microalgae have been recently analyzed [31,32,33]. In general, microalgae are characterized by (i) simplicity and standardization of growing conditions; (ii) the ability to absorb/accumulate inorganic metal ions; and (iii) economic efficiency of production. Thus, the potential of microalgae as a reliable source of nanoparticles is confirmed in a number of studies.

Despite all the advantages of microalgae as a reliable source of vesicles, only a few studies have investigated the potential of PEVs from microalgae in biomedicine. This study aimed to analyze PEVs from the culture medium of two microalgae, *Parachlorella kessleri* C15 (Chlore-EVs) and *Chlamydomonas reinhardtii* CC125 (Chlamy-EVs). The microalga *C. reinhardtii* is a well-studied model organism with simple growth conditions, the vesicles of which were isolated and characterized in a single study [28]. *P. kessleri,* a green unicellular microalga belonging to the order Chlorellales, is a poorly studied but promising biomass producer. Biotechnological application of *P. kessleri* allows, on the one hand, for obtaining a large amount of biomass and lipids in a short time, and, on the other hand, for obtaining a potentially sufficient number of extracellular vesicles for pharmaceutical use, which can be extracted simultaneously with the biomass from the culture medium. Our goal was to test the prospect of extracellular vesicles of these microalgae as delivery systems, evaluate the efficiency of their loading with exogenous biomolecules, their penetration into mammalian cells and tissues in vitro and in vivo, and also demonstrate the possibility of varying growth conditions to increase the efficiency of vesicle release in a biotechnological perspective. Additionally, we analyzed the proteome of PEVs from microalgae in order to search for potential therapeutic biomolecules in their composition.

## 2. Results

### 2.1. Characterization of Physical Parameters of Microalgae EVs

In the first step of the study, we have isolated microvesicles from the culture medium of *P. kessleri* and *C. reinhardtii*, and then we characterized the physical parameters of the vesicles: size, shape, zetta potential, and the number of vesicles obtained from a milliliter of medium.

**Figure 1:**
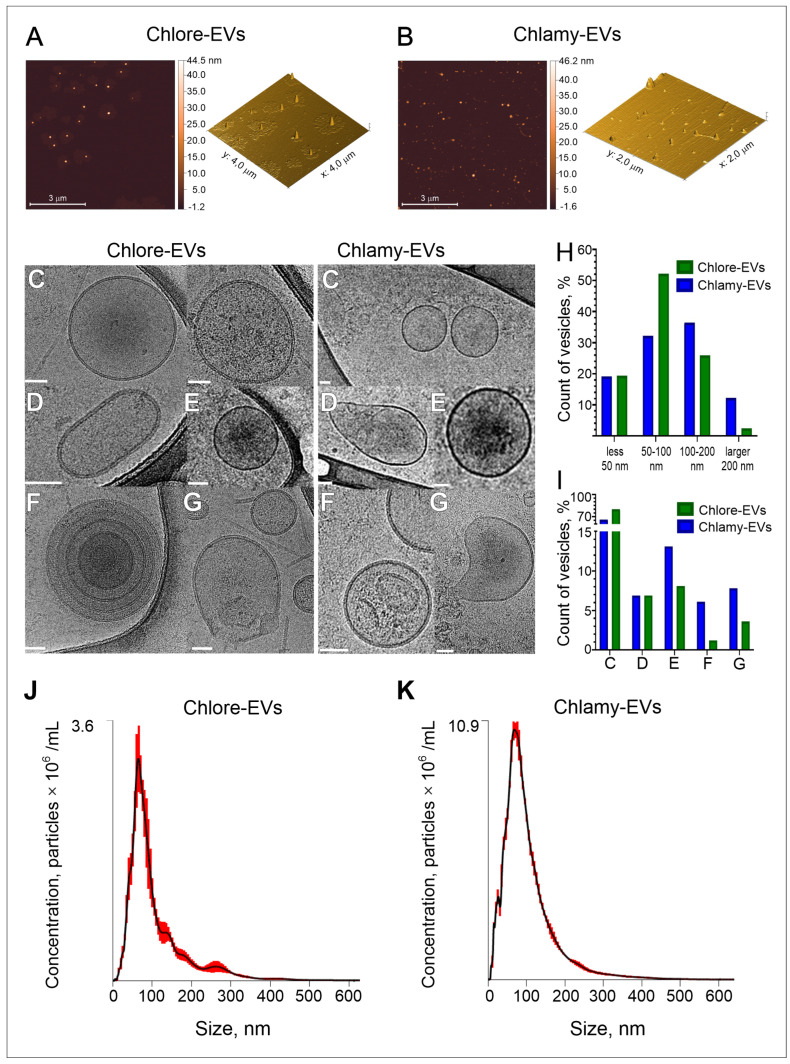
Characterization of the vesicles isolated from microalgae culture medium. (**A**,**B**) Evaluation of morphology of microalgae EVs by atomic force microscopy (AFM). The representative images of Parachlorella-derived vesicles, Chlore-EVs (**A**) and Chlamydomonas-derived vesicles, Chlamy-EVs (**B**) are presented. (**C**–**I**) Morphology and size distribution of microalgae-derived vesicles obtained by cryo-electron microscopy (cryo-EM). The representation microphotographs of EVs with various morphology: spherical single vesicles (**C**), oval-shaped vesicles (**D**), vesicles with electron-dense cargo in the lumen (**E**), multilayered vesicles (**F**), vesicles with compromised membrane integrity (**G**). Histogram of vesicle size distribution (**H**) and percentage of vesicles (**I**) with different morphology. Scale bar is 50 nm. (**J**,**K**) Examples of nanoparticle tracking analysis (NTA) of size and concentration in the isolated samples of Chlore-EVs and Chlamy-EVs.

Atomic force microscopy (AFM) was used to determine the shape and estimate the size of vesicles isolated from the microalgae culture medium. The visualization of the character landscapes of Chlore-EVs and Chlamy-EVs is shown in Figure 1A,B. It was found that most of the particles were spherical or with a stretched-out half-spherical shape due to an artifact of the method [34]. The particles of varying sizes, ranging from 10 nm to 100 nm in height, were observed in the micrographs. The most frequently encountered particles were observed between 30 and 90 nm in size both for Chlore-EVs (~90%) and Chlamy-EVs (~80%). The larger particles in the range of 90–150 nm (~10–20%) were also recorded in the preparation of Chlamy-EVs. Thus, according to AFM data, the microalgae vesicles show similar morphology and size to higher plant vesicles [35], as well as to exosomes of human biofluids [36].

Considering that AFM, due to the limitations of the method, shows only an approximate picture of the morphology and size of biological nanoparticles, cryo-electron microscopy (cryo-EM), as one of the most reliable methods, was used to determine the main parameters of the microalgae-derived extracellular vesicles. Figure 1C–I demonstrate the analysis of the morphology and size distribution of vesicles obtained from microalgae culture media by cryo-EM. A total of 270 and 100 vesicles were visualized for Chlore-EVs and Chlamy-EVs, respectively. Among vesicles analyzed, about 80% of Chlore-EVs and 65% of Chlamy-EVs were spherical in shape, surrounded by a lipid bilayer (Figure 1C,I). Oval-shaped (Figure 1D), multilayered particles (Figure 1E), as well as vesicles with increased electron density (Figure 1F) and with disrupted membranes (Figure 1G), were also observed.

In addition to the particle morphology analysis, the size of native microalgae vesicles was determined. The size distribution of Chlamy-EVs and Chlore-EVs differs significantly from the normal one; therefore, the data on vesicle sizes are presented as median (min; max).

According to the data obtained during the analysis of cryo-EM images in Image J 1.54g software [37], the size of Chlore-EVs is 71 nm (28 nm; 312 nm), and the size of Chlamy-EVs is 99 nm (24 nm; 499 nm). Among the vesicles derived from *C. reinhardtii*, the presence of the larger particles was observed. About 36% of the Chlamy-EVs had a size in the range of 100–200 nm and 12% were larger than 200 nm (Figure 1H). Thus, using the cryo-EM method, the vesicles corresponding in size and morphological characteristics to extracellular vesicles of higher plants were identified in samples isolated from the culture medium of microalgae.

The nanoparticle tracking analysis (NTA) was used as a more routine method to estimate the concentration of particles isolated from microalgae culture media. A total of 1 × 10^12^ Chlore-EVs and 0.3 × 10^12^ Chlamy-EVs were obtained from 75 mL of culture medium when the microalgae were cultured under standard conditions. The hydrodynamic particle diameter data were also obtained. It was shown that the particle sizes of around 80 nm for Chlore-EVs and around 90 nm for Chlamy-EVs were the most frequent (mode of distribution) in the samples (Figure 1J,K).

Next, to analyze the charge of the particles obtained from microalgae culture media, we determined the Z-potential of the vesicles that was equal to −11 ± 2 mV and −5 ± 2 mV for Chlore-EVs and Chlamy-EVs, respectively. The value of Z-potential, which is associated with the surface charge of particles [38], was determined for vesicles derived from different plants. These values were as follows: −15 mV for cabbage [39], −17 mV for broccoli [5], −10 mV for carrot [40], −7 mV for tea flowers [41]; for microalgae vesicles, this parameter has not been reported so far.

Summarizing the data obtained, microalgae *P. kessleri* and *C. reinhardtii* secrete the microvesicles into culture medium, mostly spherical in shape, enclosed in a lipid bilayer, possessing a negative zetta potential and around 100 nm in size. These physical characteristics are similar to exosome-like particles of higher plants and mammalian exosomes.

### 2.2. The Biocompatibility of Chlore-EVs and Chlamy-EVs In Vitro

**Figure 2:**
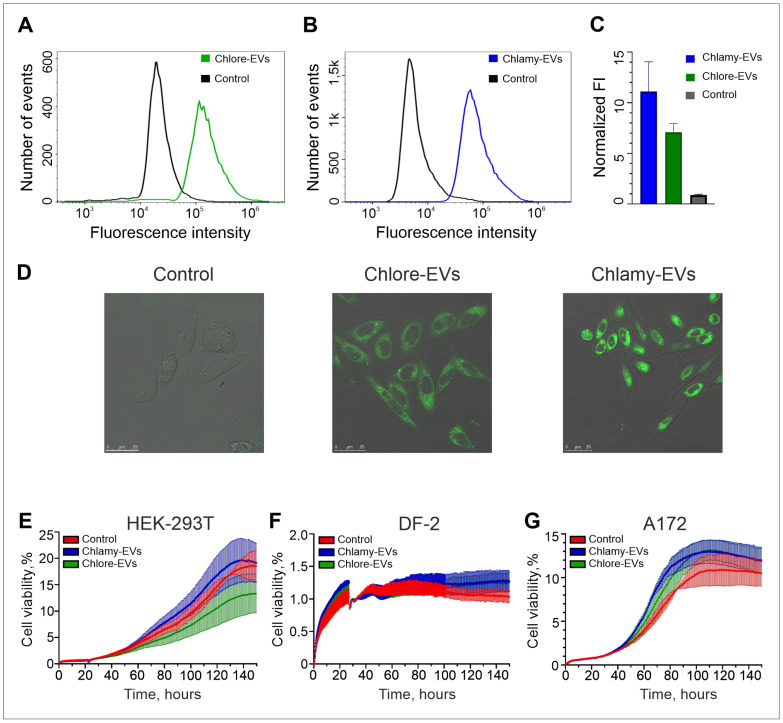
The uptake of Chlamy-EVs and Chlore-EVs by target human cells and their effect on proliferative activity of cultured human cells of normal morphology and cancer cells. (**A**–**C**) Flow cytometry analysis of LoVo cells co-incubated with Chlore-EVs (**A**) and Chlamy-EVs (**B**) labeled with BDP 493/503 lipid stain. (**C**) The estimation of cellular internalization by LoVo cells of the specific stained microalgae EVs. The experiments were performed in triplicate. (**D**) The confocal microscopy of a fluorescence signal accumulation in the cytoplasm of LoVo cells after co-incubation with fluorescently stained Chlore-EVs and Chlamy-EVs. Scale bars are 25 μm. Control (**A**–**D**)—the staining and washing procedures with BDP 493/503 were performed as in experiment but in the absence of microalgae EVs. (**E**–**G**) Examples of proliferative activity of cells with normal morphology HEK-293T (**E**) and DF-2 (**F**), as well as the glioma cell line A172 (**G**) when co-cultivated with microalgae vesicles. Analysis of cell proliferation in the presence of Chlamy-EVs and Chlore-EVs (10^6^ vesicles/cell) was carried out in real time using the xCellLigence instrument. Control cells were incubated with culture medium only. The average of two replicates is shown for each condition. Error bars represent standard deviations.

For recording the the availability of human cells to uptake the microalgae-derived EVs, Chlore-EVs and Chlamy-EVs were conjugated with a lipid stain BDP 493/503. The effective penetration of the conjugated EVs after co-incubation with human LoVo cells was confirmed in the flow cytometry experiments (Figure 2A–C). Indeed, the fluorescence intensity of LoVo cells co-incubated with the stained Chlore-EVs and Chlamy-EVs increased 7 and 11 times, respectively, as compared to a control where initial staining reaction with BDP 493/503 was washed in the absence of microalgae vesicles (Figure 2C). In addition, the accumulation of the fluorescence was registered in the LoVo cell cytoplasm 2 h after the addition of fluorescently labeled vesicles to the cells (Figure 2D).

Since microalgae vesicles can be considered as delivery systems for both exogenous and endogenous therapeutic biomolecules, it is necessary to ensure that they do not have a cytostatic effect on cells of normal morphology, and also do not stimulate the proliferation of tumor cells. The carrier of exogenous biomolecules, when effectively penetrating into cells, should not have a toxic effect on them, which, according to the ISO 10993-5:2009 standard, corresponds to at least 70% survival when the drug is added. Human embryonic kidney cells (HEK-293T) and dermal fibroblasts (DF-2) were used to analyze the effects of Chlore-EVs or Chlamy-EVs on the proliferative activity of the cells with normal morphology. Cell proliferative activity was monitored in real time using the xCellLigence system. Co-incubation of the HEK-293T and DF-2 cell cultures with Chlore-EVs and Chlamy-EVs had no adverse effects on cells, indicating their safety in systems in vitro (Figure 2E,F). In addition, vesicles from both microalgae had neither a stimulatory nor an inhibitory significant effect on the proliferative activity of A172 glioma cells (Figure 2G). All experiments to assess cell proliferative activity in the presence of microalgae vesicles were repeated three times.

### 2.3. The Biodistribution of Chlore-EVs and Chlamy-EVs In Vivo

**Figure 3:**
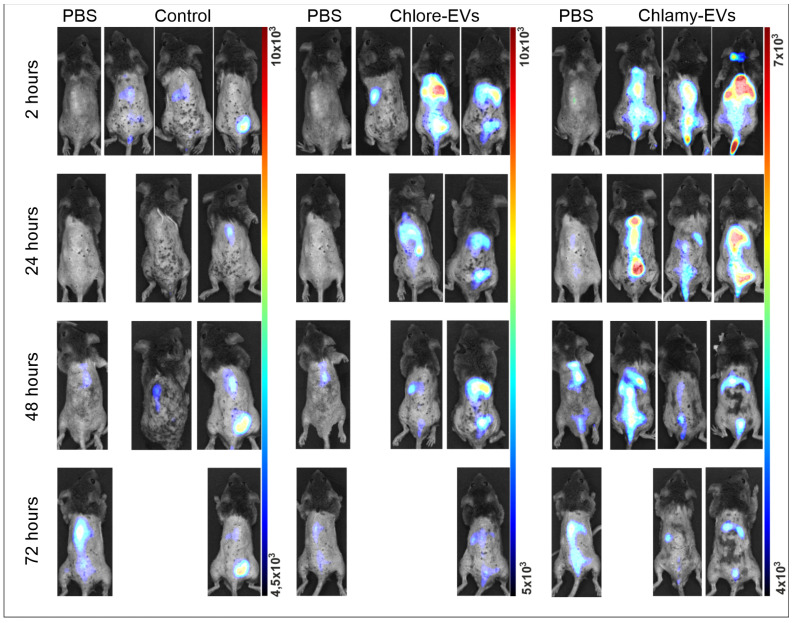
Biodistribution of Chlamy-EVs and Chlore-EVs in mice. The accumulation and distribution of fluorescence of the microalgae-derived EVs labeled with lipophilic tracer DiR in a 72 h period after tail vein injection are presented. Control mice (Control) were injected with a solution obtained as a result of identical labeling and washing procedures with DiR, but without adding microalgae vesicles, to control the washing procedure and visualize the background fluorescence of the free tracer. PBS—group for visualization of autofluorescence.

To analyze the distribution of Chlore-EVs and Chlamy-EVs in vivo, mice were injected in the tail vein with vesicles labeled with a fluorescent lipophilic tracer DiR, as well as the DiR fraction obtained by the staining and washing in the absence of EVs as a control for the specificity of fluorescence from Chlore-EVs and Chlamy-EVs. The intravital imaging revealed evidence of microalgae-derived vesicle accumulation in the peritoneal cavity and thoracic region of mice as early as 2 h after injection (Figure 3).

**Figure 4:**
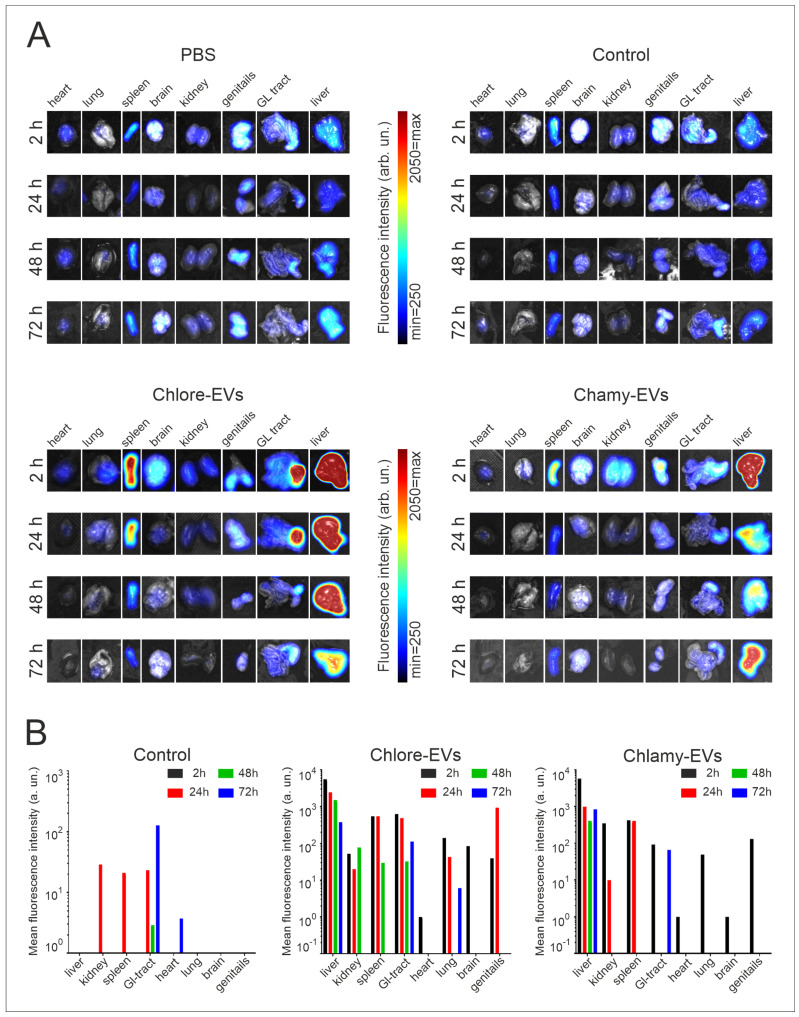
Biodistribution of DiR-labeled Chlore-EVs and Chlamy-EVs in the organs of mice. (**A**) Ex vivo visualization of fluorescence in organs at 2, 24, 48 and 72 h after tail vein injection. (**B**) Histograms represent ex vivo quantification of fluorescence signal of each organ at different time points. Control mice (Control) were injected with a solution obtained as a result of identical labeling and washing procedures with DiR tracer, but without adding microalgae vesicles. PBS—group of mice for visualization of organ autofluorescence.

At each time point, one animal per group was sacrificed and dissected, and the mean fluorescence intensity from the liver, kidney, spleen, gastrointestinal tract, genitals, brain, heart, and lungs was analyzed (Figure 4).

When Chlamy-EVs were administered, the main fluorescence signal was in the liver (5768 a.u.), and a relatively significant signal was also observed in the spleen (422 a.u.) and in the kidneys (350 a.u.). Thus, it can be assumed that the main proportion of vesicles accumulates in the liver. A minor signal was observed in the genitals, gastrointestinal tract and lungs, while no fluorescence was detected in the heart and brain, suggesting the absence of vesicles in them. A 6-fold decrease in fluorescence intensity was shown in the liver over the next 24 h, and a 14-fold decrease was seen by 48 h of observation. However, vesicles were still detected in the liver after 72 h, but the fluorescence intensity had significantly decreased. After 24 h, no signal was observed from the gastrointestinal tract, lungs, and genitals, indicating that Chlamy-EVs had been eliminated from them. The fluorescence signal in the kidney and spleen, the intensity of which was maintained at 24 h of observation, was not detected after 48 h.

When Chlore-EVs were administered, a major signal was also observed for the liver (5541 a.u.), which was identical to Chlamy-EVs. The signal intensity decreased over time, but was less pronounced for the first two days. Thus, in the first 24 h, the signal dropped by 2 times; after 48 h, the fluorescence intensity decreased by 4 times; and only on the 3rd day, the signal intensity dropped by 15 times. In the first 2 h, a relatively significant fluorescence was obtained from the spleen, gastrointestinal tract and lungs, but it was 10 and 40 times lower, respectively, than in the liver. In the spleen, the fluorescence intensity was fully preserved up to 24 h and completely ceased to be detected only on the 3rd day of observation. In the lungs, the signal was 3 times lower the next day and ceased to be significantly detected on the 2nd day of observation, and for the gastrointestinal tract, the fluorescence dropped 5 times at 72 h of observation. A 100 times less intense fluorescence was observed in the kidneys, which persisted for up to 48 h. In the genitals, the signal was registered in the first 2 h, the intensity of which increased 4 times by 24 h, but was no longer detected on the 2nd day of observation. It is also worth noting that weak fluorescence was detected for the brain in the first 2 h, which was not detected after the 1st day.

Since free lipophilic tracer DiR can bind to the cell membranes, cell compartments and tissues, thus creating a fluorescence background, a control group of mice (Control) was added to the experiment. This group was injected with a solution obtained as a result of identical labeling with DiR and washing procedures, but without adding microalgae vesicles. Thus, testing the control group of animals allowed us to evaluate both the efficiency of the washing procedure from free DiR, and the intensity of background fluorescence caused by the lipophilic tracer. In the control group of mice, the accumulation of fluorescence signal was not registered in any organ in the first 2 h after injection. At 24 h, the signal intensity in the spleen was 10 times lower than that from the administration of Chlore-EVs and Chlamy-EVs. The fluorescence detected in the kidneys and gastrointestinal tract was comparable for animals in the experimental and control groups, which may indicate the non-specificity of the signal and the elimination of vesicles from the organs in the first day after administration.

### 2.4. Microalgae Vesicles as Delivery System for Exogenous Biomolecules

**Figure 5:**
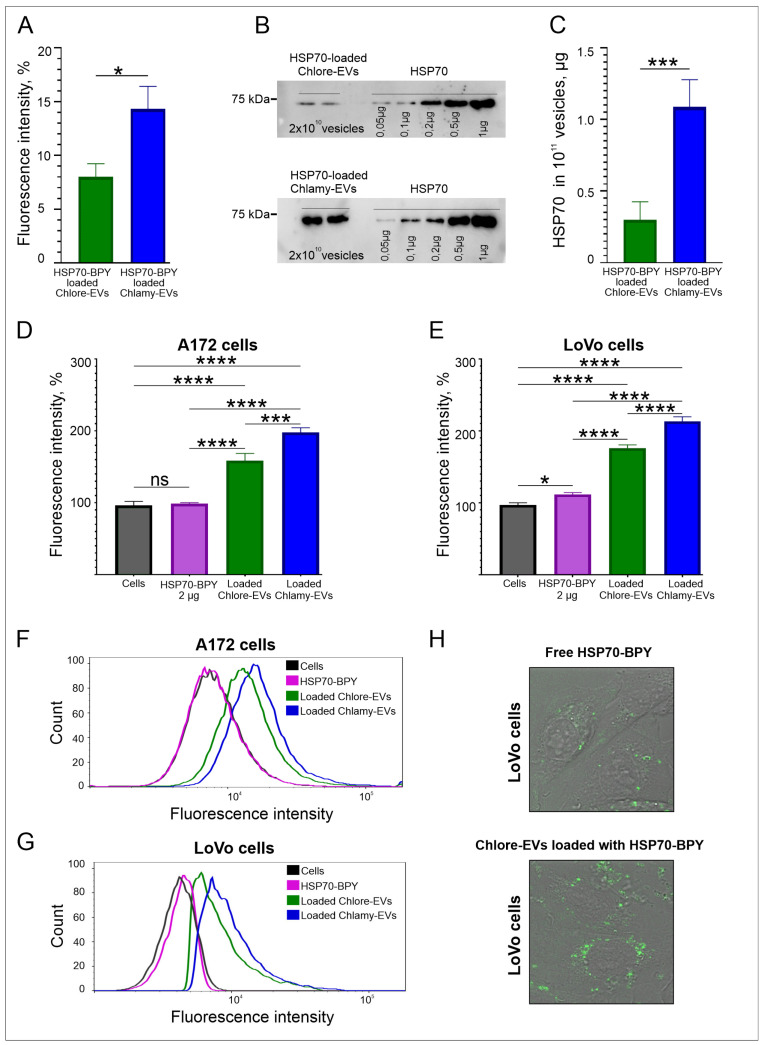
Loading and penetration of microalgae vesicles into human cells. (**A**) The efficiency of HSP70-BPY loading to Chlamy-EVs and Chlore-EVs (EVs loaded with HSP70-BPY) by fluorimetry analysis. (**B**,**C**) The efficiency of HSP70 loading to Chlamy-EVs and Chlore-EVs by Western blotting. (**B**) An example of Western blot of lysed Chlamy-EVs and Chlore-EVs, after the loading with HSP70. (**C**) The estimation of loading efficiency of HSP70 to Chlore-EVs and Chlamy-EVs. (**D**,**E**) The delivery efficiency of HSP70-BPY by Chlamy-EVs and Chlore-EVs to A172 glioma (**D**) and LoVo colon carcinoma (**E**) cells. Data were normalized relative to control. Control was taken as 100%. Pairwise multiple comparisons were performed using one-way ANOVA with Tukey’s posterior test: *p* < 0.00001 = ****, *p*< 0.0001 = ***, *p* < 0.05 = *. (**F**,**G**) Examples of flow cytometric analysis of the uptake of loaded Chlore-EVs/Chlamy-EVs vesicles, as well as free HSP70-BPY, by A172 (**F**) and LoVo (**G**) cells. (**H**). The accumulation of HSP70-BPY-loaded Chlore-EVs in the cytoplasm of LoVo cells in comparison with free fluorescent protein HSP70-BPY (2 µg) in confocal microscopy.

To study the potential of microalgae vesicles as a delivery system for exogenous molecules, Chlore-EVs and Chlamy-EVs obtained from microalgae cultural medium were loaded with a fluorescently labeled recombinant human HSP70 protein (HSP70-BPY). The integrity of most vesicles after the loading procedure was confirmed by cryo-EM and NTA (Figure S1). The loading efficiency was evaluated by fluorometry. The fluorescence signal of the initial mixture of HSP70-BPY (0.05 mg/mL) and vesicles (5 × 10^11^ particles) was taken as 100%. The fluorescence intensity of the vesicles washed from the excess of free proteins after the loading procedure was measured. The loading efficiency of Chlore-EVs and Chlamy-EVs was estimated as 8% and 16% of the starting mixture, corresponding to about 0.5 µg and 1 µg of HSP70-BPY in 10^11^ particles, respectively (Figure 5A). As a control, a loading and washing procedure was performed with the same amount of HSP70-BPY but without adding microalgae vesicles, and a negligible fluorescence was registered in the preparation, indicating a fairly effective purification of the vesicles from the free HSP70-BPY.

The loading efficiency of microalgal EVs with HSP70 was also estimated by Western blotting analysis (Figure 5B,C). Free recombinant HSP70 protein was applied on gel in the range from 0.05 µg to 1 µg, as well as HSP70-loaded Chlore-EVs and Chlamy-EVs in the amount of 2 × 10^10^ vesicles per lane (Figure 5B). It was shown that 10^11^ of Chlore-EVs contain 0.3 ± 0.1 µg of HSP70, and 10^11^ of Chlamy-EVs contain 1.1 ± 0.2 µg of HSP70 (Figure 5C). Thus, the loading efficiency of recombinant HSP70 determined in Western blot analysis is in accordance with the efficiency obtained from fluorometric data of microalgae EVs loaded with HSP70-BPY.

To estimate the efficiency of delivery of exogenous molecules into human cells, the Chlore-EVs and Chlamy-EVs loaded with HSP70-BPY were added to A172 glioma and LoVo colon carcinoma cells in the amount of 10^6^ particles/cell. Free HSP70-BPY (2 µg) was added to cells as a control. According to the evaluation of fluorescence intensity of cells by flow cytometry, the protein within vesicles accumulates more efficiently in the human cells than the free protein (Figure 5D–G). Indeed, Chlore-EVs were shown to contribute to a 1.6-fold increase in cell fluorescence, while Chlamy-EVs contributed to a 2-fold increase in cell fluorescence. The accumulation of fluorescence signal in the cytoplasm of LoVo cells detected by confocal microscopy confirms the higher uptake of HSP70-BPY-loaded Chlore-EVs by the target cells as compared to free HSP70-BPY (Figure 5H). These data demonstrate that the exogenous protein can be efficiently loaded and delivered to human cells in vitro by microalgae vesicles.

### 2.5. Microalgae Vesicles as Carriers of Endogenous Biomolecules

Mass spectrometry (LC-MS) analysis was performed to reveal the diversity of endogenous protein molecules carried by Chlore-EVs and Chlamy-EVs. The protein samples of vesicle lysates were verified using the Bradford colorimetric test and SDS-PAGE analysis with Coomassie R-250 staining (Figure S2).

**Figure 6:**
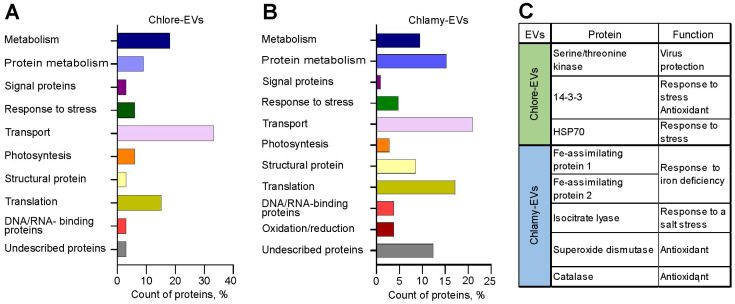
Analysis of the protein composition of microalgae-derived vesicles. (**A**,**B**) Molecular functions of proteins identified in Chlore-EVs (**A**) and Chlamy-EVs (**B**). (**C**) The proteins providing plant defense against stress and exhibiting antioxidative activity identified in Chlore-EVs or Chlamy-EVs.

A total of 33 proteins for Chlore-EVs and 105 for Chlamy-EVs were identified (see Tables S1 and S2). Among them, 6 proteins were common to EVs secreted by both microalgae: Glyceraldehyde-3-phosphate dehydrogenase, ATP synthase gamma chain, proteasome subunits alpha and beta, ribulose bisphosphate carboxylase large chain, and histone H4. To investigate the functional basis of the identified proteins from microalgae EVs, we have analyzed and classified their molecular functions. The annotated biological functions of the proteins revealed an enrichment of the microalgae vesicle-associated proteins related to “transport”, “protein metabolism”, “translation”, and “metabolism” (Figure 6A,B).

Figure 6C shows proteins with described functions providing plant responses to different types of abiotic and biotic stress and exhibiting antioxidative activity detected in EVs. Among the contents of Chlore-EVs are protein 14-3-3, a regulator of plant metabolism and stress responses [42], serine/threonine kinase, which is associated with the defense of plants against viruses and pathogens [43], and heat shock protein 70 (Hsp70). Moreover, Chlamy-EVs revealed abundant Fe-assimilating protein 1 (FEA1) and Fe-assimilating protein 2 (FEA2) involved in the microalgae’s response to iron deficiency. *FEA1* gene encodes an iron-assimilation protein presumably functioning as an iron chaperonin that delivers iron to metal transporters [44]. Isocitrate lyase, identified in Chlamy-EVs, plays an important role in plant salt tolerance [45]. In addition, such antioxidant proteins as superoxide dismutase [46] and catalase [47] were identified in Chlamy-EVs (Figure 6C).

### 2.6. The Effect of the Microalgae Cultivation Conditions on the Yield of Secreted Vesicles

Next, we tested the possibility of increasing the specific yield of vesicles by varying the conditions of the producer culture. Chlore-EVs and Chlamy-EVs collected under different microalgae growth conditions were isolated from 75 mL of culture medium by ultracentrifugation. The concentrated vesicle pellets were diluted in 500 μL of PBS and analyzed by NTA to estimate the number of the particles obtained.

#### 2.6.1. The Variability of Cultivation Conditions for *P. kessleri*

**Figure 7:**
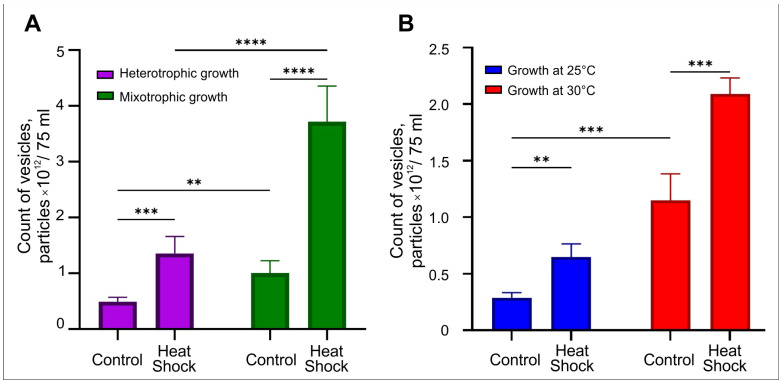
NTA analysis of vesicles produced from 75 mL of culture medium of the microalgae under varying culture conditions. (**A**) Estimation of the amount of Chlore-EVs produced from a culture medium of *P. kessleri* under mixotrophic and heterotrophic growth with heat shock. (**B**) Evaluation of the amount of Chlamy-EVs produced from a culture medium of *C. reinhardtii* at 25 °C and 30 °C with heat shock. Pairwise multiple comparisons were performed using one-way ANOVA with Tukey’s posterior test: *p* < 0.00001 = ****, *p* < 0.0001 = ***, *p* ≤ 0.005 = **.

Mixotrophic (TAP medium with 1% glucose under illumination) and heterotrophic growth (TAP medium with 1% glucose without illumination) were used for culturing of *P. kessleri*, with and without heat shock treatment. The results of NTA analysis of Chlore-EV preparations obtained from *P. kessleri* culture medium under different conditions are shown in Figure 7A. The heterotrophic growth reduces the number of particles obtained at least 2 times compared to mixotrophic conditions. Exposure to heat shock in mixotrophic and heterotrophic type of growth increases the yield of particles by 3.6 and 2.7 times, respectively. Thus, the highest specific yield of vesicles from the culture medium of *P. kessleri* was obtained with mixotrophic growth and a heat shock treatment.

#### 2.6.2. The Variability of Cultivation Conditions for *C. reinhardtii*

*C. reinhardtii* was incubated in TAP medium at 25 °C and 30 °C, and the microalgae were exposed to heat shock. Figure 7B illustrates the results of NTA, reflecting the dependence of vesicle secretion by microalgae on the temperature of incubation and heat shock parameter. The increase in growth temperature from 25 °C to 30 °C raised the yield of vesicles more than 3-fold. At the same time, the exposure to heat shock leads to a more- than-2-fold increase in the number of vesicles released at both temperatures. Thus, the highest number of particles was produced by *C. reinhardtii* cultured at 30 °C and exposed to heat shock. As a result, the variation in growth conditions allowed for increase in the specific yield of vesicles by 7.3 times.

## 3. Discussion

In recent years, plant-derived vesicles have attracted increasing attention in terms of their potential use in biomedicine. Although microalgae can be a promising producer of exosome-like particles, only a small number of works are devoted to vesicles derived from unicellular algae. In the present study, we describe extracellular vesicles purified from the culture medium of two microalgae: *C. reinhardtii* CC125 and *P. kessleri* IPPAS C15.

*C. reinhardtii* is a classic model organism in cellular and molecular biology. It is widely used to study photosynthesis, flagellar motility, cell cycle, and chloroplast biology. The genetic accessibility, a sequenced genome, and a large collection of mutants make it an important model for microalgae research [48]. Meanwhile, *P. kessleri* is a less established model organism, but has attracted attention due to its physiological robustness, high productivity, and potential for industrial applications, especially in lipid and biomass production [49,50,51]. *P. kessleri* is a unicellular eukaryotic green alga known for its high growth rate and efficient photosynthetic activity. It is well adapted to various stressful environmental conditions, including nitrogen deficiency and salt stress. From a biotechnological perspective, both species of producers are promising and have different physiological features, which may have their own advantages depending on the demand. Thus, *P. kessleri* is characterized by stress resistance and productivity, and *C. reinhardtii* is genetically controllable and has been extensively studied to date.

Microalgae-derived Chlore-EVs and Chlamy-EVs were characterized following MISEV2023 recommendations [52]. The microalgae particles in native form were visualized for the first time by cryo-EM high-resolution imaging as one of the most accurate methods [53,54], which allowed us to detect predominantly spherical-shaped vesicles with a lipid bilayer, which is also characteristic of higher plant [16] and human vesicles [55,56].

The size of the vesicles obtained in this work were estimated by different methods with limitations in each; however, the determined size values appeared to be similar and were within the same ranges as the sizes of vesicles of other plant-derived nanoparticles studied to date (Table 1).

According to cryo-EM analysis, the median size of Chlore-EVs and Chlamy-EVs was about 71 nm and 99 nm, respectively. The NTA data showed a main population of particles with a mode of 80 nm for Chlore-EVs and 90 nm for Chlamy-EVs, which correlated with cryo-EM results.

In the present work, we demonstrated that microalgae vesicles belong to negatively charged particles (−11 mV for Chlore-EVs, and −5 mV for Chlamy-EVs). The Z-potential value is an important characteristic of extracellular vesicles [38], especially when they are used as delivery vehicles for therapeutic drugs. The negatively charged nanoparticles show lower protein adsorption on their surfaces, which implies a higher chance of penetration through the blood–brain barrier or less impact from blood plasma proteins when delivered through the bloodstream [57], and such particles are more efficiently taken up by human cells [58].

**Table 1:** Characteristics of plant-derived extracellular vesicles (PEVs) from different sources.

Source	Species	Average Size	Z-Potential	Refs
Bitter melon	*Momordica charantia*	100–200 nm	−	[59]
Cabbage	*Brassica oleracea* var. *capitata*	100 nm	−15 mV	[39]
Lemon	*Citrus × limon*	50–70 nm	−	[60]
Broccoli	*Brassica oleracea* var. *italica*	32 nm	−17 mV	[5]
Strawberry	*Fragaria × ananassa*	30–100 nm	−	[11]
Carrot	*Daucus carota* subsp. *sativus*	150 nm	−10 mV	[40]
Tea flowers	*Camellia sinensis*	131 nm	−7 mV	[41]
Glaucophyte	*Cyanophora paradoxa*	130 nm	−	[31]
Chlorophyte	*Tetraselmis chuii*	100 nm	−	[32]
Chlorophyte	*Dunaliella tertiolecta*	88 nm	−	[61]
Chlorophyte	*Haematococcus pluvialis*	89 nm	−	[62]
Chlorophyte	*Parachlorella kessleri*	71 nm	−11 mV	This work
Chlorophyte	*Chlamydomonas reinhardtii*	99 nm	−5 mV	This work

Moreover, our study demonstrates the possibility to use microalgae vesicles (*C. reinhardii* and *P. kessleri*) as carriers of therapeutic exogenous biomolecules. The vesicles of plant origin, including microalgae-derived vesicles, are a natural product of extraction and have some advantages over synthetic particles, which currently have a few unresolved problems in the field of their use in nano-pharmacological delivery systems [63,64].

The plant-derived vesicles have a natural shell, which facilitates their uptake by the recipient cells. This assumption has been experimentally confirmed in several studies. Thus, effective penetration into mammalian cells on various cellular models was shown for cabbage-derived vesicles on HaCaT cells (human keratinocyte cell line) [39], apple-derived vesicles on Caco-2 cells (human colorectal adenocarcinoma cells) [65], and *T. chuii*-derived nanoalgosomes on the MDA-MB-231 cells (human breast adenocarcinoma) [33], and grapefruit-derived nanovesicles were efficiently captured by the A549 cells (human lung carcinoma) and CT-26 cells (mouse colon carcinoma) [6]. Also, the natural origin contributes to a reduction in cytotoxic effects on cells of normal morphology, as reflected previously in [39], where the addition of cabbage-derived vesicles did not result in cytotoxic effects on HaCaT, HDFs (normal human primary dermal fibroblasts), and RAW264.7 cells (macrophage-like cell line). In study [66], colon-26 epithelial-like cells and RAW 264.7 cells were co-incubated with ginger-derived vesicles, and HNF (normal skin cells) cells were co-incubated with vesicles derived from *Dendropanax morbifera* [67]. The absence of cytotoxic effects on cells of normal morphology was also demonstrated in the present work, where it was shown that HEK-293T and DF-2 had no effect on cell viability in real time over several days, confirming the biocompatibility of the microalgae-derived vesicles as drug carriers.

The microalgae nanovesicles labeled with a fluorescent lipophilic tracer DiR were used to analyze their biodistribution in mice. Similarly to other nanoparticles studied, both Chlore-EVs and Chlamy-EVs demonstrated the accumulation in liver and spleen in the first 2 h after injection. According to cryo-EM and NTA data, a major fraction of particles in Chlore-EV and Chlamy-EV samples are less than 100 nm and possess a negative surface charge, which is associated with an increased half-life and elimination of nanoparticles from the body. It is known that noncontinuous endothelia with vascular fenestrae in the liver have a size of 50–100 nm [68], and the size range of interendothelial cell slits in the spleen is 200–500 nm [69]. Thus, particles larger than 100 nm can accumulate in the liver, and those larger than 200 nm in the spleen. The accumulation of vesicles in the liver and spleen may also indicate their phagocytosis by the reticuloendothelial system.

Based on our results, we can assume that Chlore-EVs are more stable in the animal organism, since when Chlore-EVs were administered, the fluorescence signal was detected in the genitals, lungs, gastrointestinal tract, spleen and liver after 24 h, in the spleen, kidneys, gastrointestinal tract and liver after 48 h, and in the lungs and liver after 72 h of observation. However, the fluorescence signal was registered only in the spleen and the liver at 24 h after the injection of Chlamy-EVs. Thus, Chlore-EVs can be noted to be sufficiently stable in the body, which will ensure their effective accumulation in organs and tissues when used as a platform for the delivery of therapeutic biomolecules.

Despite the growing interest in plant vesicles for their use in biomedicine, only a few studies have provided information on the biodistribution of PEVs [18,60,70]. Thus, in the work of X. Ou et al. [70], an analysis of the distribution of vesicles derived from *Catharanthus roseus* was presented using different routes of administration. When administered intravenously, accumulation of the majority of vesicles was observed in the liver and spleen, and a small portion of vesicles were also detected in the lungs. The ginger-derived lipid nanoparticles designed by M. Zhang et al. [18] accumulated in the spleen, kidneys, liver, heart, and lungs after intravenous administration. In a study by S. Raimondo and colleagues [60], accumulation of lemon vesicles in the liver, spleen, and kidneys was shown after 24 h of intraperitoneal administration.

Thus, our data on the biodistribution of Chlore-EVs and Chlamy-EVs correspond to the previously presented results, and, as in the studies of X. Ou [70] and M. Zhang [18], accumulation of a portion of the vesicles in the lungs was detected, which can probably be retained by the pulmonary capillaries, and, as in the work of M. Zhang [18], accumulation of a small portion of the vesicles in the heart was observed. Additionally, our study demonstrated accumulation of Chlore-EVs and Chlamy-EVs in the brain during the first 2 h, as well as in the genitals during the first 24 h.

The obtained data indicate sufficient stability of Chlamy-EVs and Chlore-EVs in the body and a long period of elimination, which allows the vesicles to accumulate in tissues and organs, while this type of vesicles is characterized by accumulation in the liver and spleen, similar to nanoparticles of various natures [71]. For the first time, we have shown that microalgal vesicles can potentially pass the blood–brain barrier and accumulate in the brain, but the results require validation in further studies.

The possibility of using plant vesicles as delivery vehicles for therapeutic drugs has been previously evaluated. In studies [19,72], ginger- and grapefruit-derived vesicles were used to create liposomal particles that were loaded with methotrexate and applied to treat DDS-induced colitis in animal models. A similar method was investigated in [61], where vesicles from the microalga *Dunaliella tertiolecta* were also reconstructed into liposomal particles, and their ability to transport a model drug was studied.

The use of native plant vesicles for delivery has also been investigated previously. The possibility of using the Survivin siRNA-loaded ginger-derived vesicles for in vivo tumor therapy in a mouse model of nasopharyngeal adenocarcinoma was demonstrated [12]. In several other studies authors used the cabbage-derived vesicles loaded with doxorubicin for antitumor therapy on the SW480 cell model [39], or evaluated the antitumor activity of 5-Fluorouracil-loaded bitter melon-derived vesicles in a murine and oral carcinoma cell delivery model [73], or demonstrated the possibility of using grapefruit vesicles to deliver exogenous proteins to human cells [16]. Recombinant heat shock protein 70 has demonstrated a high ability to induce an antitumor immune response and is considered as an adjuvant for the creation of antitumor vaccines [21]. In a series of previous studies, we demonstrated the possibility of using exosomes and vesicles from grapefruit juice to deliver the functionally active chaperone HSP70 to mammalian cells in vitro and significantly increase its bioavailability and antitumor effect in an animal model of colorectal cancer [16,20,22]. In the present study, we confirmed that therapeutic recombinant human HSP70 can be efficiently loaded to microalgae vesicles and successfully delivered into human cells in vitro.

In this study, we also analyzed the composition of endogenous proteins of the obtained Chlamy-EVs and Chlore-EVs. It should be noted that microalgae vesicles are not characterized by the rich protein constituents. Most of the proteins are related to metabolism and transport, but some proteins were identified that are associated with the response of plants to different types of abiotic and biotic stress, which may provide further evidence that one of the main functions of such vesicles in plants is defense against pathogens [74].

We compared the microalgae vesicle proteomes with the proteomes of vesicles obtained from strawberries [75], tomato fruits [76,77], citrus fruits (sweet orange (*C. sinensis*), lemon (*C. limon*), grapefruit (*C. paradisi*), bitter orange (*C. aurantium*)) [78], *Nicotiana tabacum* [79], and *Arabidopsis thaliana* [80]. Common proteins among all the considered producers that were found in the content of Chlamy-EVs and Chlore-EVs are mainly ribosomal proteins, histone proteins, elongation factors, and proteasomal subunits. Common antioxidant proteins such as superoxide dismutase and catalase in Chlamy-EVs with vesicles of strawberries, tomato fruits, and citrus fruits should be separately noted. The other antioxidant protein 14-3-3 was detected in Parachlorella vesicles, and was found in vesicles of strawberries [75], tomato [76,77] and citrus fruits [78], *N. tabacum* [79] and *A. thaliana* [80], as well as heat shock protein 70 (HSP70), which was previously considered as a marker of plant vesicles [33]. Also, the enzyme Glyceraldehyde-3-phosphate dehydrogenase, responsible for glycolysis, carboxylic anhydrase, related to photosynthesis, or Citrate synthases, involved in the Krebs cycle, was found in all vesicles studied.

Because of the data on the functionality of major and common proteins, as well as the presence of antioxidant proteins among them, we can assume that vesicles of both microalgae and higher plants can be involved in similar biogenesis processes and, moreover, can be used as antioxidative agents. The antioxidative activity has been shown both for plant extracts and their vesicles [7,10,11]. Taking into account that antioxidative activity has also been shown for microalgal extracts [29], vesicles from *C. reinhardtii* and *P. kessleri*, similarly to the above-mentioned particles of other plants, may have antioxidative activity and be used in biomedicine.

All of the above suggest that microalgal vesicles can be used to deliver therapeutic biomolecules to human cells without cytotoxic effects on the cells of a normal morphology. Notably, the main advantage of microalgae vesicles as carriers of therapeutic biomolecules is their technological production. The possibility of cultivating microalgae under constant controlled conditions suggests obtaining vesicles with identical standard characteristics. The main disadvantages of microalgae vesicles include the low yield of particles isolated from one liter of culture medium, compared, for example, with the number of particles isolated from one liter of juice of edible plant fruits [16,17,39,81]. We have demonstrated here that the varying the conditions can increase the specific yield of particles up to seven times, which is definitely an important factor for biotechnology. The obtained data open up prospects for finding optimal conditions for cultivating microalgae for biotechnological production of vesicles with maximum yield.

## 4. Materials and Methods

### 4.1. Microalgae Source and Cultivation

*Parachlorella kessleri* IPPAS C15 was obtained from the collection of Timiryazev Institute of Plant Physiology RAS (Moscow, Russia). *Chlamydomonas reinhardtii* CC125 (*137c nit1 nit2 agg1þ mtþ*) was obtained from the Chlamydomonas Resource Center, RRID: SCR_014960; http://www.chlamycollection.org (accessed on 17 April 2024).

*P. kessleri* cells were cultured mixotrophically in tris-acetate phosphate (TAP) medium containing 1% glucose at 30 °C, with circular rotation at 120 rpm in a shaker-incubator (INFORS HT, Bottmingen, Switzerland), with light. *C. reinhardtii* cells were grown in TAP medium in glass flask, under agitation at 120 rpm on orbital shaker (ELMI, Riga, Latvia) at room temperature (25 °C) and under constant illumination with white LED at 5000 K. Both microalgae cultures were initiated with starting concentration of ~10^6^ cells/mL. The culture growth was monitored by measuring optical density at 600 nm and 750 nm for Parachlorella and Chlamydomonas strains, respectively.

### 4.2. Variation of Microalgae Cell Culture Conditions

Microalgae culture conditions were modified for optimization of the yield of secreted vesicles. The *P. kessleri* cells were cultured in TAP medium containing 1% glucose at 30 °C mixotrophically with light or heterotrophically in the absence of light. The *C. reinhardtii* was cultivated at two different temperatures, 25 °C and 30 °C. To test the effect of heat shock treatment on a specific yield of EVs secreted by *P. kessleri* and *C. reinhardtii*, the microalgae cells were cultured under standard conditions for 7 days; then the culture flasks were placed in a thermal shaker and incubated for 1 h at 40 °C, after which the cells were further cultured for 24 h under standard conditions until collection of the culture medium. All experiments were carried out at least three times at different periods of the year, and at least five identical samples were used in each experiment.

### 4.3. Isolation of EVs

Isolation of vesicles from the culture medium of microalgae was carried out using the method of isolating vesicles from fruits of higher plants [16,17] with some modifications. The microalgae culture media were centrifuged at 1500× *g* for 15 min at 8 °C to separate cells from the culture medium. Then, supernatant was centrifuged at 3000× *g* for 20 min, at 10,000× *g* for 1 h and at 16,000× *g* for 1 h at 8 °C to remove large debris. The first low-speed centrifugation steps were performed using JA-10 rotor for Avanti J-25I Centrifugation. EVs were isolated in 75 mL tubes for TYPE 45Ti rotor for Optima L-90K Ultracentrifuge at 150,000× *g* for 2 h at 8 °C. Then, the pellet was incubated in 1 mL PBS for 15 h at 4 °C, re-suspended in PBS, and centrifuged again at 16,000× *g* for 1 h at 8 °C. Then EVs were re-isolated by ultracentrifuge at 150,000× *g* for 2 h at 8 °C in 5 mL tubes for SW55Ti rotor for Optima L-90K Ultracentrifuge. The pellet was incubated in 50 μL PBS for 15 h at 4 °C. Then, the pellet was re-suspended in PBS and frozen at −80 °C.

### 4.4. Atomic Force Microscope (AFM)

Surface topology of microalgae vesicles was estimated by AFM. Before the measurements, EV samples were further cleared of coarse debris by centrifugation at 13,000× *g* for 1 h at 8 °C. For analysis, 5 μL of EVs at a concentration of 10^10^ particles/mL were deposited onto freshly cleaved mica (TipsNano, Tallin, Estonia). After complete drying at RT, the samples were washed with miliQ to remove salt crystals and dried at RT for 15 h. The sample topography measurements were performed in semi-contact mode using the atomic force microscope “NT-MDT-Smena B” with a NSG03 probe (NT-MDT, Zelenograd, Russia). The images were analyzed using Gwyddion 2.4 software [82].

### 4.5. Cryogenic Electron Microscopy (Cryo-EM)

The morphology of EVs was assessed by cryo-electron microscopy using a Titan Krios 60–300 TEM/STEM transmission cryo-electron microscope (FEI, Hillsboro, OR, USA) equipped with a highly sensitive Falcon II electron detector (FEI) and a spherical aberration corrector (CEOS, Heidelberg, Germany). For analyses, EV samples (3 μL) were applied to copper grids coated with a thin layer of amorphous carbon, followed by freezing the samples in liquid ethane (−196 °C). As a result, EVs fixed in a layer of amorphous ice were obtained, which made it possible to evaluate the morphology of EVs in the native state.

### 4.6. Nanoparticle Tracking Analysis (NTA)

The concentration and size of EVs were detected by nanoparticle tracking analysis (NTA, Malvern Instruments, Malvern, Worcestershire, UK) using a NanoSight^®^ LM10 analyser (Malvern Instruments, Malvern, Worcestershire, UK) with a violet laser (45 mW at 405 nm) and a C11440-5B camera (Hamamatsu photonics K.K., Iwata, Shizuoka, Japan). Measurements were performed at 25 °C with the given parameters of camera intensity—16, threshold from 0 to 2015 (detection 7–9), minimum expected particle size—30 nm. The video recording of Brownian motion of EVs with duration of 30 s was performed three times. The results were analyzed using NTA 2.3 software.

### 4.7. Z-Potential

The Z-potential of EVs was estimated by electrophoretic light scattering also using a Photocor Compact-Z (Photocor LLC, St. Petersburg, Russia) equipped with a red laser (25 mV, 640 nm). During sample preparation, 20 μL of particle suspension with a concentration of 10^12^ particles/mL was diluted in 2 mL of milli-Q water and centrifuged at 12,000 rpm for 40 min to precipitate large scatterers. Measurements were performed for 90 s at 25 °C nine times.

### 4.8. Cell Lines

Cell lines of normal morphology and human malignant neoplasms of different localization were used in this work. The glioma cell line (A172) and colon carcinoma (LoVo) were used for experiments to evaluate the efficiency of penetration of microalgae vesicles loaded with exogenous protein HSP70. Human embryonic kidney cells (HEK-293T) and dermal fibroblasts (DF-2) were used to analyze the effects of algae EVs on the proliferative activity of cells of normal morphology. Cell lines were obtained from the collection of Institute of Cytology of the Russian Academy of Sciences (St. Petersburg, Russia). Cells were cultured in DMEM/F12 medium containing L-glutamine (Biolot, St. Petersburg, Russia), with 10% fetal bovine serum (BioWest, Nuaillé, France) and the antibiotic gentamicin at a final concentration of 0.1 mg/mL. The cells were incubated at 37 °C and 5% CO_2_.

### 4.9. Fluorescence Labeling of Chlore-EVs and Chlamy-EVs with Lipofilic Tracers

Two lipophilic fluorescence tracers were used for labeling of algae EVs. Chlore-EVs and Chlamy-EVs were conjugated with lipid stain BDP 493/503 (Lumiprobe, Moscow, Russia) for experiments on the internalization of EVs by human cells in vitro. The lipophilic tracer DiR (Lumiprobe, Moscow, Russia) was used for biodistribution analysis of algae EVs in vivo. For the staining, 10 µg of BDP 493/503 or DiR was slowly added to the vesicles at a concentration of 10^12^ particles/mL to a final concentration of 35 µg/mL, followed by co-incubation at 37 °C for one hour. The mixture was adjusted to 1.5 mL by PBS and was centrifuged three times at 16,000× *g* for 15 min until the visible pellet disappeared. Then, the stained vesicles were washed again using 100 kDa filters (Amicon, Merck, Darmstadt, Germany) to remove excess dye, and finally centrifugated twice at 16,000× *g* for 15 min. The samples obtained after the same staining and washing procedures with BDP 493/503 or DiR but in the absence of microalgae EVs were used as a control.

### 4.10. Analysis of the Penetration of Chlore-EVs and Chlamy-EVs into Colorectal Cancer Cell Line LoVo In Vitro

The LoVo cells were seeded into a 12-well plate at 10^5^ cells/well 24 h before the start of the experiment. On the next day, BDP493/503-labeled Chlore-EVs and Chlamy-EVs were added in each well in an amount of 2 × 10^5^ vesicles per cell and co-incubated for 4 h. The cells were then washed with PBS and removed with Versene-Trypsin solution, followed by analysis of fluorescence intensity using CytoFlex flow cytometry (Beckman Coulter, Brea, CA, USA). The experiments were carried out in triplicate.

For confocal microscopy analysis, LoVo cells were pre-seeded on µ-Dish Petri dishes (Ibidi, Gräfelfing, Germany) in the amount of 10^5^ cells per dish. Then, BDP493/503-labeled Chlore-EVs and Chlamy-EVs were added to the cells in the amount of 1.2 × 10^11^ and 4 × 10^10^ vesicles, respectively. After 2 h of co-incubation, LoVo cells were scanned layer by layer using a confocal microscope (LEICA TCS SP5X, Leica Microsystems, Wetzlar, Germany).

### 4.11. Cell Proliferative Activity

The effect of algae EVs on cell proliferative activity was assessed using xCELLigence RTCA system (Agilent, Santa Clara, CA, USA). Cells were seeded in special E-plates (Agilent, USA) in the amount of 5000 cells/well for cell impedance measurement. Cells were incubated for 24 h at 37 °C in a 5% CO_2_ atmosphere; then, Chlore-EVs or Chlamy-EVs were added at an amount of 10^6^ particles/cell with a complete change in nutrient medium. The change in cell impedance was analyzed during 100–150 h. Experiments were performed in duplicate.

### 4.12. Biodistribution of DiR-Labeled Chlore-EVs and Chlamy-EVs In Vivo

BALB/c mice from the vivarium of the preclinical and clinical research center of the NRC “Kurchatov Institute”-PNPI (Gatchina, Russia) were used in this study. All experiments with laboratory animals were performed in compliance with the legislation of the Russian Federation and the rules of bioethics. All manipulations were conducted in compliance with the protocol approved by the Local Bioethics Commission of the National Research Center “Kurchatov Institute”—PNPI, Commission decision No. 25/05/19/4 dated 19 May 2025. The study was carried out in compliance with the ARRIVE guidelines [83]. The 20 male BALB/c mice were randomly divided into 4 groups of 5 mice. The mice in control groups were given a single intravenous injection of PBS for determining autofluorescence of organs and tissues, and a control sample of free tracer DiR for testing the washing procedure and visualizing the background fluorescence. The experimental groups were injected with DiR-labeled Chlore-EVs and Chlamy-EVs at 4 × 10^10^ and 10^11^ vesicles, respectively. The animals were visualized in vivo using the universal optical fluorescence and luminescence visualization system LumoTrace (Abisense, Sirius, Russia) at 2, 24, 48, and 72 h after the injection. The bioimaging system used was equipped with a Retina Lumo camera (photometry) using Micromanager [84] software in Icy [85] with Abisense plugins. Fluorescence was recorded with a 50 s exposure and 6 × 6 binning.

At each time point, one animal from the group was dissected, with subsequent detection of fluorescence intensity of the spleen, liver, heart, gastrointestinal tract, genitals, brain, and kidneys also using the LumoTrace visualization system (Abisense, Sirius, Russia). Filming was performed for 20 s with 6 × 6 binning. The average fluorescence intensity for each organ was analyzed in the Icy 2.4.3.0 software, after which the average fluorescence intensity of the corresponding organ of the group with PBS injected was subtracted from the obtained values, taking as autofluorescence.

### 4.13. Labeling of Recombinant Human HSP70 with BODIPY

To modify the amino group of the HSP70 with BODIPY FL sulfosuccinimide ester, SSE (Invitrogen, D6140, Waltham, MA, USA), the recombinant human HSP70 (140 µM final) was incubated with an excess of BODIPY-FL, SSE (1 mM) in 100 mM NaHCO_3_ buffer (pH 8.5) for 1 h at 25 °C. The labeled HSP70-BPY was purified from BPY excess by gel filtration with a chromatograph Akta start #2 (GE, Uppsala, Sweden) and UNICORN start 1.01 software using an Econo-Column chromatography column (Bio-Rad, Hercules, CA, USA). Fractions containing fluorescently labeled protein were concentrated using 50 kDa centrifuge filters (Amicon, Darmstadt, Germany). The concentration of HSP70-BPY protein samples was estimated using a NanoDrop spectrophotometer (Thermo Scientific, Waltham, MA, USA).

### 4.14. Loading of Chlore-EVs or Chlamy-EVs with Recombinant Human HSP70-BPY Protein

The EVs were loaded with fluorescently labeled HSP70-BPY protein by passive loading and sonication treatment. For this purpose, HSP70-BPY at a final concentration of 0.05 mg/mL was mixed with Chlore-EVs/Chlamy-EVs at a final concentration of 1.5 × 10^12^ particles/mL and incubated overnight at 4 °C. Then, the mixture was sonicated at a frequency of 35 kHz for 15 min by the Bandelin SONOREX SUPER ultrasonic bath (Bandelin Electronic GmbH & Co. KG, Berlin, Germany) at room temperature, and incubated for additional 90 min at 4 °C. The loaded EVs were purified from free protein using centrifuge 100 kDA filters (Amicon, Merck, Darmstadt, Germany). Filtration and washing were performed at least 5 times.

Loading efficiency of Chlore-EVs or Chlamy-EVs with labeled proteins, as well as the efficiency of washing the vesicles from free protein, was analyzed by measuring the fluorescence of the samples with a spectrofluorometer (Hitachi F-7000, Hitachinaka, Ibaraki, Japan). The fluorescence intensity of the initial mixture of vesicles and protein at a concentration of 0.05 mg/mL was also evaluated and taken as 100%. The experiments were carried out twice and included three FI measurements of each sample.

The efficiency of Chlore-EVs/Chlamy-EVs loading with recombinant HSP70 was also assessed by Western blotting. The samples of algae-derived vesicle lysates obtained from 2 × 10^10^ particles were separated on a 10% SDS-PAGE and transferred onto a methanol-activated PVDF membrane (Thermo Scientific, Thermo Scientific, Waltham, MA, USA) using the Trans-Blot Turbo Transfer System (Bio-Rad, Hercules, CA, USA). The staining was carried out by applying the specific mouse monoclonal antibodies to HSP70 (clone 8D1, patent # Ru2722398, Russia) for 1 h, after which the secondary HRP-Linked Rabbit Anti-Mouse IgG Polyclonal Antibody (CLOUD-CLONE CORP, Wuhan, China) was applied to the membrane. The proteins were visualized using the Clarity Western ECL Blotting Substrate (Bio-Rad, Hercules, CA, USA) on a ChemiDoc System (Bio-Rad, Hercules, CA, USA) in chemiluminescence mode.

### 4.15. The Penetration Efficiency of Chlore-EVs and Chlamy-EVs Loaded with HSP70-BPY by Human Cells In Vitro

Flow cytometry was used to evaluate the efficiency of delivery of exogenous HSP70-BPY to human cells in vitro by Chlore-EVs/Chlamy-EVs. Glioma cells A172 and colon carcinoma LoVo cells were seeded on 12-well plates at a density of 10^5^ cells/well. In order to deliver the exogenous proteins, purified samples of HSP70-BPY protein-loaded Chlore-EVs/Chlamy-EVs were co-cultured with the recipient cells. The number of loaded vesicles was determined by NTA and the equivalent number of vesicles was added to cells (10^6^ vesicles/cell). As a comparative control, cells were co-incubated with free HSP70-BPY protein added to additional wells of the plate in an amount equivalent to the amount loaded into the vesicles. After 4 h of incubation, the cells were trypsinized and collected and then washed three times with PBS and analyzed by Flow cytometry. Cell fluorescence intensity was analyzed using a CytoFlex flow cytometer (Beckman Coulter, Brea, CA, USA) in the FITC fluorescence channel, dialing a minimum of 20,000 events. The experiments were carried out twice and included three FI measurements of each sample.

### 4.16. Preparation of a Total Protein Extract of Algae-Derived Vesicles

An amount of 100 µL of extraction buffer (7 M urea, 2 M thiourea, 2% CHAPS, 0.3% DTT) was added to the algae-derived vesicle samples (10^11^, 3 × 10^11^, 10^12^ particles), and the mixture was transferred on ice for 30 min. The protein concentrations were measured with a Quick Start™ Bradford Protein Assay (Bio-Rad, Hercules, CA, USA) using the EnSpire™ Multilabel Plate Reader (PerkinElmer, Shelton, CT, USA). A minimum of 100 µg of total protein extract was used for mass spectrometry analysis. To analyze the protein profile of algae-derived vesicle lysates, the samples obtained from 10^11^ and 3 × 10^11^ particles were applied on 10% SDS-PAGE followed by Coomassie R-250 staining.

### 4.17. Mass Spectrometry Analysis

The proteomic analysis procedure was based on proteolytic hydrolysis by STrap (Suspension Trapping) [86] followed by identification of the resulting peptides by high-performance liquid chromatography with mass spectrometric detection (HPLC-MS). HPLC-MS was performed using an Ultimate 3000 RSLC nano system (Thermo Scientific, Thermo Scientific, Waltham, MA, USA) coupled to a Q-executive HFX mass spectrometer using an Acclaim µ-Precolumn enrichment column (0.5 mm × 3 mm, 5 μm particle size, Thermo Scientific Thermo Scientific, Waltham, MA, USA) at a flow rate of 10 μL/min for 4 min in isographic mode and separating HPLC column Acclaim Pepmap C18 (75 μm × 150 mm, 2 μm particle size, Thermo Scientific, Waltham, MA, USA) in gradient elution mode.

The proteins were identified using SearchGUI v.3.2.20 software with simultaneous use of search algorithms, OMSSA, MS-GF+ [87], and Uniprot database with restriction by the type of organism under study.

### 4.18. Statistical Data Processing

The statistical data analysis was performed using GraphPad Prism 9.5.1 software. Flow cytometry data processing and visualization were carried out using the freely available FlowJo_V10 plugin. Error bars in other histograms indicate the standard deviation for at least three independent experiments. Data in the histograms were compared using the nonparametric Mann–Whitney U test or one-way ANOVA followed by Tukey’s HSD test. Values of *p* < 0.05 were accepted as statistically significant differences.

## 5. Conclusions

Based on the data obtained, microalgae vesicles can be considered as protein delivery systems for biomedical applications. Firstly, these vesicles contain natural protein components with potential therapeutic properties. Thus, antioxidant protein-containing vesicles of *C. reinhardtii* can be considered natural antioxidants. Secondly, the microalgae vesicles can be used to deliver exogenous therapeutic proteins into human cells without cytotoxic or cytostatic effects on cells of normal morphology due to their high efficiency of uptake by human cells in vitro and their ability to penetrate and accumulate in tissues and organs in vivo. Moreover, the ability to influence the specific yield of vesicles by varying producer culture conditions will make it possible to obtain a biotechnologically and economically favorable producer of drug delivery agents in the future.

## Data Availability

Data are contained within the article and Supplementary Materials.

## Supplementary Materials

The following supporting information can be downloaded at: https://www.mdpi.com/article/10.3390/plants14152354/s1, Figure S1: Morphology and size distribution of Chlamydomonas-derived vesicles, Chlamy-EVs (A) and Parachlorella-derived vesicles, Chlore-EVs (B) after loading procedure with HSP70. Left panels: Morphology of microalgae-derived vesicles obtained by cryo-electron microscopy. Scale bars are 50 nm. Right panels: A typical example of nanoparticle tracking analysis (NTA) of size and concentration in the sample of microalgae EVs before and after loading procedure; Figure S2: SDS-PAGE of microalgae vesicle proteins: Chlore-EVs (A) and Chlamy-EVs (B). Samples obtained from 10^11^ and 3 × 10^11^ particles were applied; Table S1: Proteins identified from purified EVs of *Parachlorella kessleri* C15; Table S2: Proteins identified from purified EVs of *Chlamydomonas reinhardtii* CC125.

## Abbreviations

AFM, atomic force microscopy; NTA, nanoparticle tracking analysis; Cryo-EM, cryogenic electron microscopy; EVs, extracellular vesicles; PEVs, plant extracellular vesicles; MVBs, multivesicular bodies.
