# Supplementary figures and images for: Microalgae-Derived Vesicles: Natural Nanocarriers of Exogenous and Endogenous Proteins
Source: Plants (Basel). 2025 Jul 31;14(15):2354. doi: 10.3390/plants14152354 (PMC12348454; doi:10.3390/plants14152354)

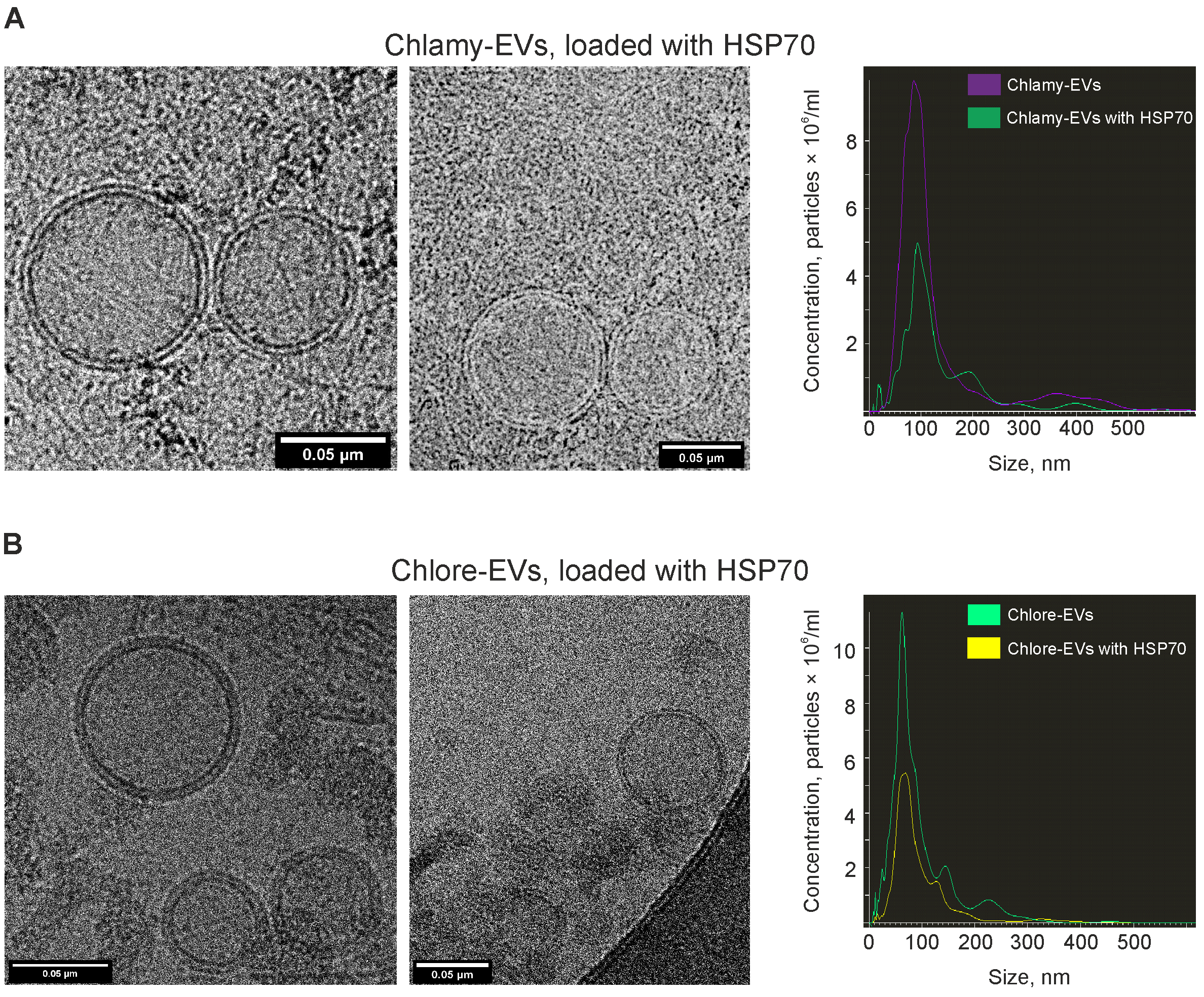

Supplement: Supplementary file 1 [file plants-14-02354-s001.zip › Figure S1.tif]

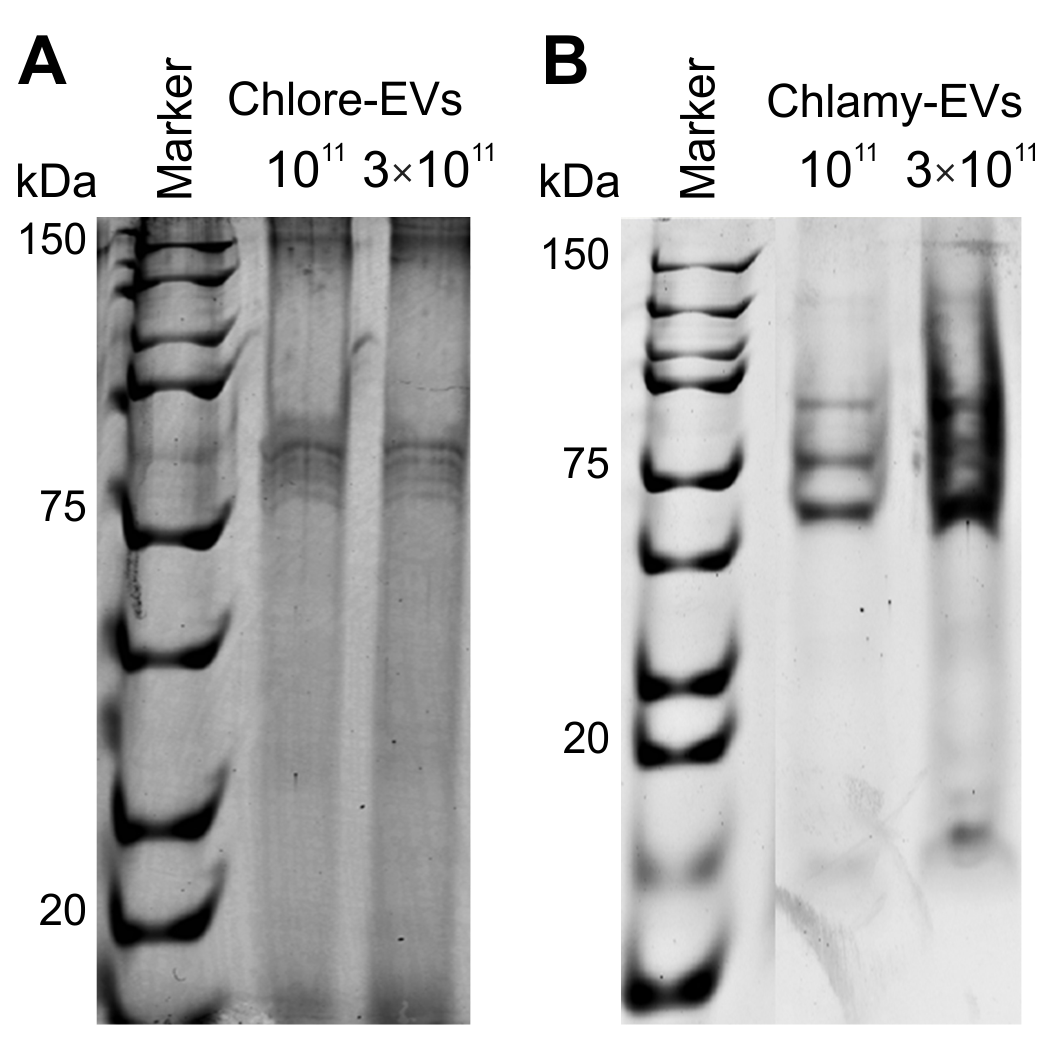

Supplement: Supplementary file 1 [file plants-14-02354-s001.zip › Figure S2.tif]
